# Anti-EGFR targeted monoclonal antibody isotype influences anti-tumor immunity in head and neck cancer patients

**DOI:** 10.1186/2051-1426-3-S2-P316

**Published:** 2015-11-04

**Authors:** Sumita Trivedi, Raghvendra M Srivastava, Fernando Concha-Benavente, Tatiana M Garcia-Bates, Jing Li, Robert L Ferris

**Affiliations:** 1 University of Pittsburgh Cancer Institute, Pittsburgh, PA, USA; 2 Department of Immunology, University of Pittsburgh, Pittsburgh, PA, USA

## 

EGFR is frequently overexpressed on several cancers, and two targeted antibodies are FDA approved but differ by isotype. Cetuximab (IgG1 isotype) has been shown to be effective at both inhibiting downstream signaling of EGFR and activating anti-tumor, cellular immune mechanisms. While panitumumab (IgG2 isotype) can inhibit downstream EGFR signaling similar to cetuximab, panitumumab might also induce antibody-dependent cell cytotoxicity (ADCC) or adaptive immunity. We sought to investigate the cellular immunity specifically activated by cetuximab or panitumumab showing that both mAb primarily activate NK cells, although cetuximab was significantly more potent than panitumumab. We also observed that although panitumumab may activate monocytes through the CD32 (FcγRIIa) receptor, neither mAb activated monocytes sufficiently to mediate ADCC. Cetuximab enhanced DC maturation to a greater extent than panitumumab, corresponding with improved cross presentation of tumor antigen by cetuximab compared with panitumumab. Indeed, improved adaptive immune responses with increased EGFR-specific cytotoxic CD8^+^ T cells were present in patients treated with cetuximab compared to those treated with panitumumab. These results suggest that although panitumumab effectively inhibits EGFR signaling to a similar extent as cetuximab, it is less effective at mediating anti-tumor, cellular immune mechanisms which may be crucial for effective therapy for HNSCC.

